# Antigenic and Genetic Diversity of Human Enterovirus 71 from 2009 to 2012, Taiwan

**DOI:** 10.1371/journal.pone.0080942

**Published:** 2013-11-15

**Authors:** Yuan-Pin Huang, Tsuey-Li Lin, Ting-Han Lin, Ho-Sheng Wu

**Affiliations:** 1 Center for Research, Diagnostics and Vaccine Development, Centers for Disease Control, Ministry of Health and Welfare, Taipei, Taiwan, Republic of China; 2 School of Medical Laboratory Science and Biotechnology, Taipei Medical University, Taipei, Taiwan, Republic of China; University of Illinois at Chicago, United States of America

## Abstract

Different subgenogroups of enterovirus 71 (EV-71) have caused numerous outbreaks of hand, foot, and mouth disease worldwide, especially in the Asia-Pacific region. During the development of a vaccine against EV-71, the genetic and antigenic diversities of EV-71 isolates from Taiwan were analyzed by phylogenetic analyses and neutralization tests. The results showed that the dominant genogroups had changed twice, from B to C and from C to B, between 2009 and 2012. The subgenogroup B5 (B5b cluster) was dominant in 2008-2009 but was replaced by subgenogroup C4 in 2010-2011. From the end of 2011 to 2012, the re-emerging subgenogroup B5 (B5c cluster) was identified as the dominant subgenogroup of EV-71 outbreaks, and subgenogroups C2 and C4 were detected in sporadic cases. Interestingly, the amino acid substitution at position 145 in the VP1 gene was observed in some strains isolated from patients with acute flaccid paralysis. Furthermore, thirty-five strains and their corresponding serum samples were used to analyze the cross-protections and antigenic diversities among different subgenogroups (C4a, C5, B4, B5b, B5c, and C2-like) of EV-71. Evident antigenic diversity existed only for the C2-like subgenogroup, which was not effectively neutralized by other serum samples. In contrast, the anti-C2-like serum sample showed broad cross-reactivity against all other subgenogroups. Therefore, these results may provide valuable information for the selection of EV-71 vaccine candidates and the evolution of EV-71 subgenogroups in Taiwan from 2009 to 2012.

## Introduction

As a member of the genus *Enterovirus* of the family *Picornaviridae*, human enterovirus 71 (EV-71) is one of the most prevalent pathogens infecting young children and may cause outbreaks of hand-foot-mouth disease (HFMD), herpangina, and severe neurological complications [1]. More than one hundred serotypes have been identified in the genus *Enterovirus* [2], and classified into twelve species. EV-71 and other 23 serotypes are now members of the species *Enterovirus A* (http://www.picornaviridae.com/enterovirus/enterovirus.htm).

The EV-71 genome possesses approximately 7,500 nucleotides and encodes four structural capsid proteins (VP4, VP2, VP3, and VP1), and seven nonstructural proteins (2A, 2B, 2C, 3A, 3B, 3C, and 3D). Untranslated regions are located at both ends of the EV-71 genome. The capsid proteins contain antigenic sites that correspond to virus serotyping and receptor binding [1,3,4]. In previous studies, the VP1 protein was identified to induce human EV-71-specific CD4^+^ T-cell proliferation and was capable of eliciting neutralizing antibodies against EV-71 [5,6]. In addition, a VP1 mutation was shown to be the determinant of mouse adaptation and virulence [7,8]. Generally, sequencing of VP1 has been used for genotyping and phylogenetic analysis, and three genogroups (A, B, and C) were classified in EV-71, although the use of a combination of VP1 and 3D RNA polymerase gene sequences was also proposed for initial genotyping of EV-71 isolates [9]. Genogroup A contains only one strain, the prototype strain BrCr, while genogroups B and C are each divided into five subgenogroups (B1-B5 and C1-C5, respectively) [10,11]. Furthermore, some rare genogroups were identified sporadically, including subgenogroups B0, C0, D, and C2-like [9,12-15]. Although molecular biological methods have generally been used for the rapid detection and characterization of EV, virus isolation is still considered the gold standard in EV identification, and neutralization tests are used for serotyping and antigenic grouping.

EV-71 was first isolated in California in 1969, and it has been responsible for several outbreaks in different countries, most recently in Cambodia [16]. Since the discovery of genogroup A in the USA, that prototype strain was recently reported in China in 2008 [17]. Subgenogroups B1 and B2 circulated in the USA, Japan, Australia, and other countries between 1970 and 1990 [12,18]. Subgenogroups B3 to B5 have been described in Australia and the Asia-Pacific region since 1997 [10,19,20]. Subgenogroups C1 and C2 have been dominant in the USA and Europe since 1980s, C3 was first described in Korea in 2000, and C4 and C5 have been observed in the Asia-Pacific region since 1997 [10,11,14,21-23]. These epidemic patterns show that one subgenogroup may circulate in the same region for a long period (e.g., subgenogroup C4 in China) or cause outbreaks in different countries (e.g., subgenogroup C1 in the USA and Europe).

In 1998, a large HFMD outbreak caused by subgenogroups C2, B4, and C4 was reported in Taiwan. Subsequently, the subgenogroup B4 resulted in major outbreaks from 1999 to 2003, followed by subgenogroup C4 from 2004 to 2005, and subgenogroup B5 in 2008 [14,24,25]. In addition, a small number of subgenogroups, the C5, C4 and C2-like strains, were also observed in 2008 [14]. A rapid change of subgenogroups in Taiwan resulted in the possibility of severe outbreaks. Because of disease control and vaccine development needs, we analyzed genetic and antigenic diversity of the EV-71 isolates collected by the surveillance system from 2009 to 2012 and of reference strains by phylogenetic analyses and neutralization tests.

## Materials and Methods

### Ethics Statement

The study was approved by the Institutional Review Board (IRB) of Taiwan Centers for Disease Control (Taiwan CDC). All virus strains isolated from patients in this study were de-identified according to the IRB approved protocol of Taiwan CDC to meet the patient confidentiality guidelines. Based on the Taiwan’s Communicable Disease Control Act, an informed consent is not necessary for collecting clinical specimens from patients with suspected notifiable communicable diseases. However, if these samples were used for research purposes, they must be de-identified prior to publication. In the study, the EV-71 virus strains have been de-linked from patient identifiers.

### Specimen collection, virus isolation and identification

The EV-71 viruses used in this study were collected and isolated by the surveillance systems under the Taiwan CDC, as previously described [14]. Throat swabs, rectal swabs, or stool samples taken from patients with HFMD, herpangina, and other symptoms related to EV infection were used for virus isolation. The isolates were then identified by immunofluorescence assay (IFA) using commercial antibodies against enteroviruses (pan-enterovirus) and specifically against EV-71 (Light Diagnostics, Millipore Corporation, Billerica, MA, USA) according to the protocol recommended by the manufacturer. The cell culture infective dose (CCID_50_) of the virus was calculated by the Reed and Muench method [26].

### Sequencing and phylogenetic analysis

Viral RNA was extracted using a QIAamp Viral RNA Mini Kit (Qiagen, Santa Clara, CA) according to the manufacturer’s instructions. One-step RT-PCR of the VP1 gene was performed with primer set 159/162 [27], and full-length RT-PCR was performed as previously described [28]. The products were then sequenced using a BigDye Terminator v3.1 Cycle Sequencing Kit and an automated sequencer ABI 3730 (Applied Biosystems, Foster City, CA, USA). 

The Basic Local Alignment Search Tool (BLAST) and Molecular Evolutionary Genetics Analysis (MEGA) program were used for genotyping by sequence comparisons with reference sequences in GenBank [29,30]. Phylogenetic trees were constructed by the MEGA program, using the neighbor-joining method with a bootstrap value of 1,000. The trees were drawn to scale proportional to evolutionary distances. The genetic distances were computed using the Tamura-Nei method and were in the units of the number of base substitutions per site. The rate variation among sites was described by a gamma distribution. The differences in the composition bias among sequences were considered in evolutionary comparisons. All positions containing alignment gaps and missing data were eliminated from the dataset. The prototype coxsackievirus A16 G-10 strain was used as an out-group. Recombination analysis of full-length genome sequences was performed by using SimPlot software, as previously described [19,31,32]. A substitution map was generated based on the entropy values of amino acid sequences with a selection criterion of 5%. Different types of amino acids were indicated by different colors with single-letter abbreviations.

### Determination of neutralization antibody titers

To determine neutralization antibody titers, serum samples and isolates from 35 EV-71-infected patients representing different subgenogroups were used, including B4 (8 isolates), B5b (7 isolates), B5c (3 isolates), C4a-1 (11 isolates in 2004-2005), C4a-2 (4 isolates in 2010-2011), C5 (1 isolate), and C2-like subgenogroup (1 isolate). First, the sera were inactivated at 56°C for 30 min; then, they were serially diluted two-fold with DMEM. Next, the diluted antisera were mixed with one hundred CCID_50_ viruses (50 μl) of different subgenogroups (B4, B5b, B5c, C4a-1, C4a-2, C5, and C2-like) and placed in a CO_2_ incubator at 36°C for 60 min. One hundred μl of RD cell suspension containing approximately 3 x 10^4^ cells was subsequently added to each well, and the cytopathic effects were recorded daily for the next 4 days. The neutralization titers were defined as the highest dilution fold at which the cytopathic effects were inhibited in 50% of cells. All determinations were performed in duplicate.

### Statistical analysis

The neutralization antibody titers were log_2_ transformed and compared between the C2-like group and other subgenogroup groups. Statistical analyses were performed using GraphPad Prism 5 software (GraphPad Software, San Diego, CA). The p value < 0.05 is taken to indicate statistically significance.

### Nucleotide sequence accession numbers

The 33 nucleotide sequences identified in this study were deposited in the GenBank database under the following IDs: KF134454-KF134486.

## Results

### Epidemiological and BLAST results of EV-71 isolates

The number and location of sampling sites for collecting specimens, and the laboratory tests for diagnosis of enteroviruses were not changed during the investigation. From 2009 to 2012, a total of 22,575 specimens were collected and cultured for virus isolation, in which 10,775 enterovirus isolates were confirmed by IFA. The isolation rates of enterovirus each year during this period were 42.4% (2,440/5,749), 42.2% (3,181/7,533), 57.7% (3,060/5,302), and 52.5% (2,094/3,991), respectively. Among them, there were 961 EV-71 isolates in Taiwan used for further study, including samples from 373 females and 588 males. No significant differences were observed in gender distribution (p>0.05). The patients ranged in age from 1 week to 97 years, and most (758/961, 78.9%) of them were under 5, including 286 females and 472 males.

**Figure 1:**
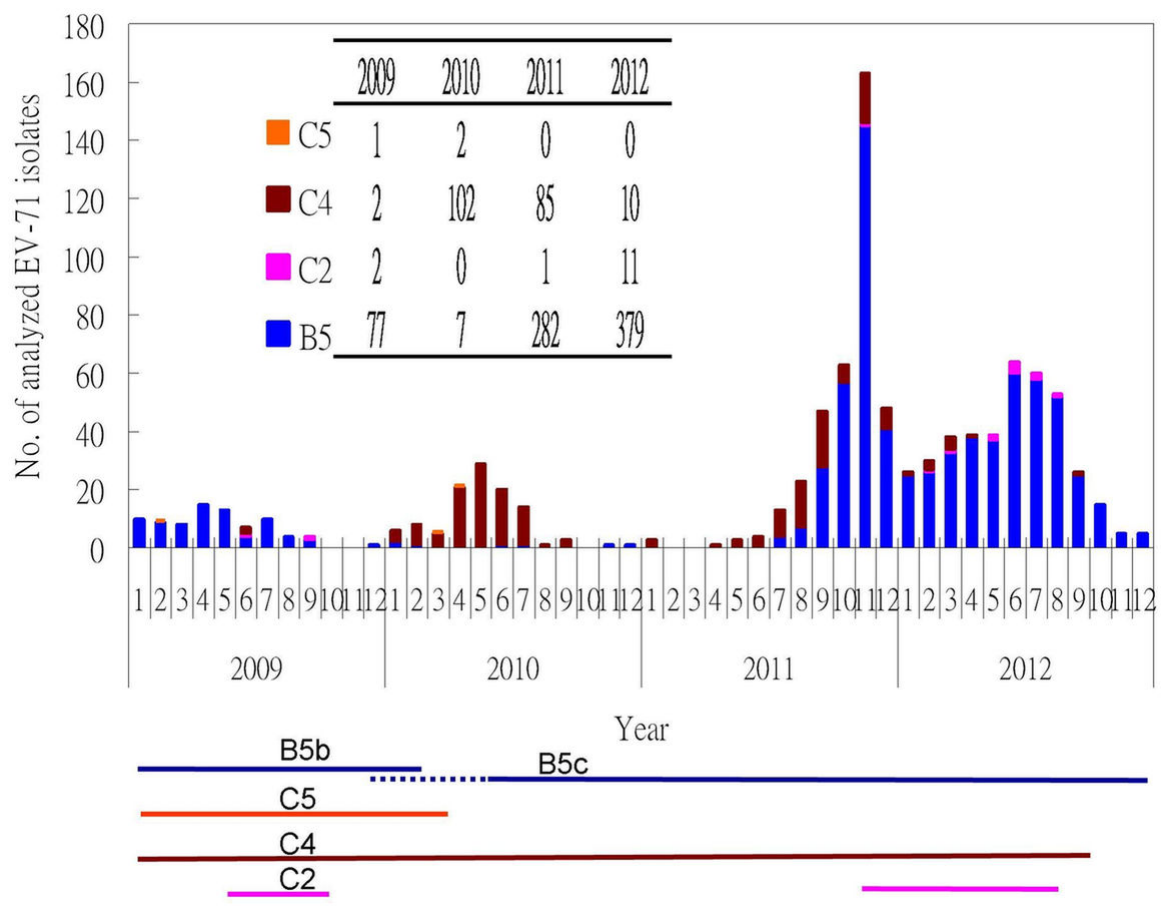
Subgenogrouping of enterovirus 71 (EV-71) isolates from Taiwan from 2009 to 2012 according to the BLAST results. The subgenogroup was determined by BLAST analysis of partial nucleotide sequences of the VP1 gene. There were 745 subgenogroup B5 isolates, 14 subgenogroup C2 isolates, 3 subgenogroup C5 isolate, and 199 subgenogroup C4 isolates identified according to the BLAST analysis.

Four subgenogroups of EV-71, including 745 subgenogroup B5 isolates, 14 C2 isolates, 199 C4 isolates, and 3 C5 isolates, were identified based on the BLAST results of partial nucleotide sequences of the VP1 gene (Figure 1). All isolates showed extremely high identities with their respective reference strains (>95%). Subgenogroup B5 was the dominant type in 2009, 2011, and 2012, while C4 was the dominant type in 2010. In addition, no subgenogroup C5 isolates had been identified since 2011.

### Phylogenetic analysis

**Figure 2:**
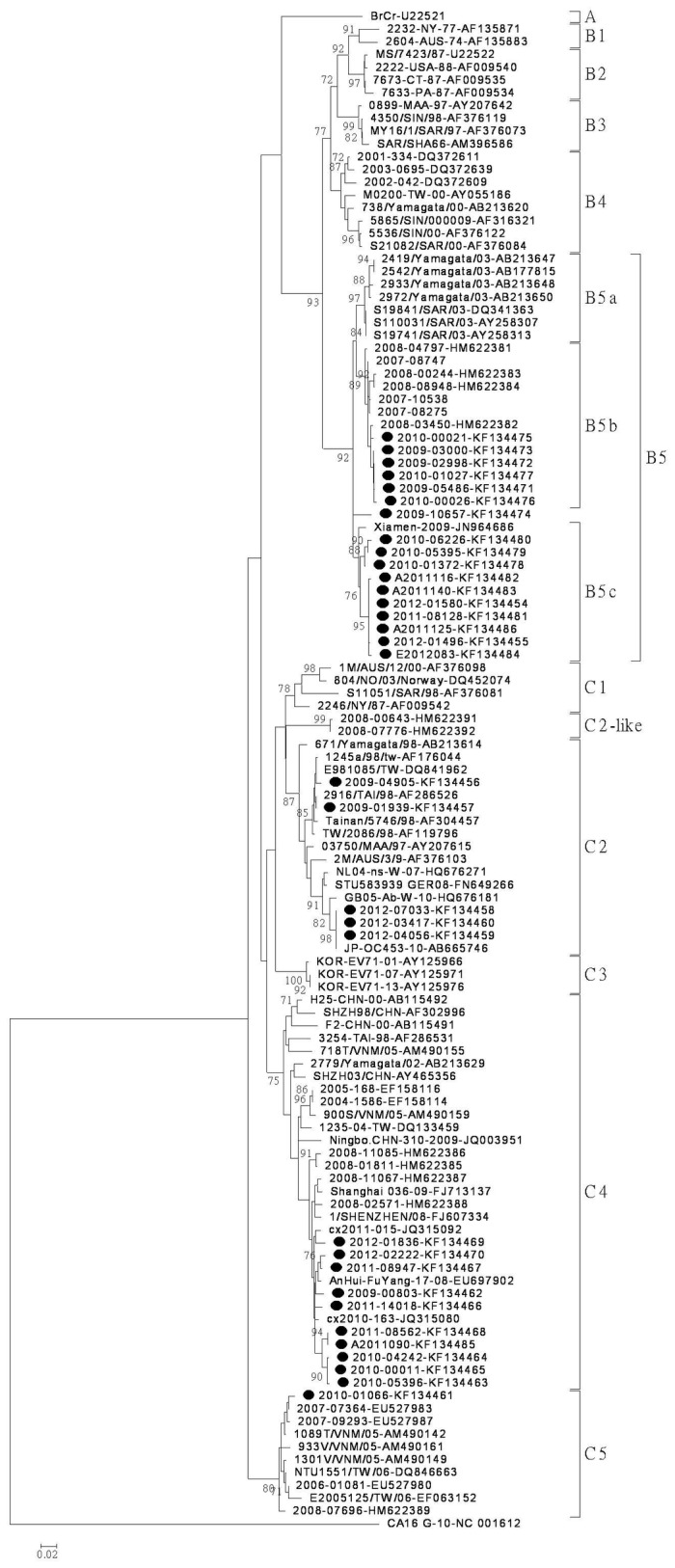
Phylogenetic analysis of enterovirus 71 (EV-71) based on partial nucleotide sequences of the VP1 gene (nucleotide position 16-418). Phylogenetic analysis was performed based on the partial nucleotide sequences of the VP1 gene of reference strains and Taiwanese isolates. The phylogenetic tree was constructed with MEGA software by the neighbor-joining method with 1,000 bootstrap replications. Only bootstrap values greater than 70% were shown.

The selection criteria of EV-71 isolates for phylogenetic analysis were 1) to represent the predominant circulating subgenogroup each year; 2) to exhibit unique characteristics; and 3) to contain all subgenogroups from 2009 to 2012. Based on BLAST genotyping results, 33 Taiwanese EV-71 isolates collected from 2009 to 2012 in this study (17 subgenogroup B5 isolates, 5 subgenogroup C2 isolates, 10 subgenogroup C4 isolates, and 1 subgenogroup C5 isolate) and reference strains recorded in the GenBank were chosen for further phylogenetic analysis on a partial VP1 gene nucleotide sequence (403 bp, nucleotide position 16–418) (Figure 2). The subgenogroup C4 isolates obtained in this study were genetically similar to each other ranging from 96.5 to100% and shared a similarity of 96.5-97.7% with the Taiwanese C4 strains isolated in 2008, which were thought to be imported from China. The C5 subgenogroup isolate was clustered with previous Taiwanese isolates and reference strains. In addition, the subgenogroup C2 isolates were clustered with different reference strains; the isolates in 2009 were clustered with older Taiwanese strains from 1998, but the isolates from 2012 were clustered with strains isolated in Japan and Europe from 2007 to 2010. Interestingly, almost all subgenogroup B5 isolates were divided into three clusters, B5a, B5b and B5c. The genetic distances from averaging over all sequence pairs within each cluster were determined as 0.009, 0.010 and 0.014 for B5a, B5b and B5c, respectively. The B5a cluster was composed of isolates from Japan and Malaysia. The Taiwanese isolates from 2009 (except one isolate 2009-10657) and some from 2010 were clustered with the strains isolated from 2007 to 2008 (B5b). The other isolates from 2010, the isolates from 2011 to 2012, and one Chinese strain from 2009 were clustered alone (B5c). The isolate 2009-10657 could not be grouped within these three clusters.

### Sequence variation and recombination analysis

To compare the genetic variations among the B5a, B5b and B5c isolates, the full-length genome sequence of one subgenogroup B5c isolate (A2011125), and the VP1 gene sequences of 15 subgenogroup B5 isolates were determined. In the recombination analysis of full-length genome sequences, no recombination event was observed for the B5c isolate (data not shown), and there was a high similarity (96.6%) of the full-length genome nucleotide sequences between the B5b (2007-08747) and B5c (A2011125) isolates.

**Figure 3:**
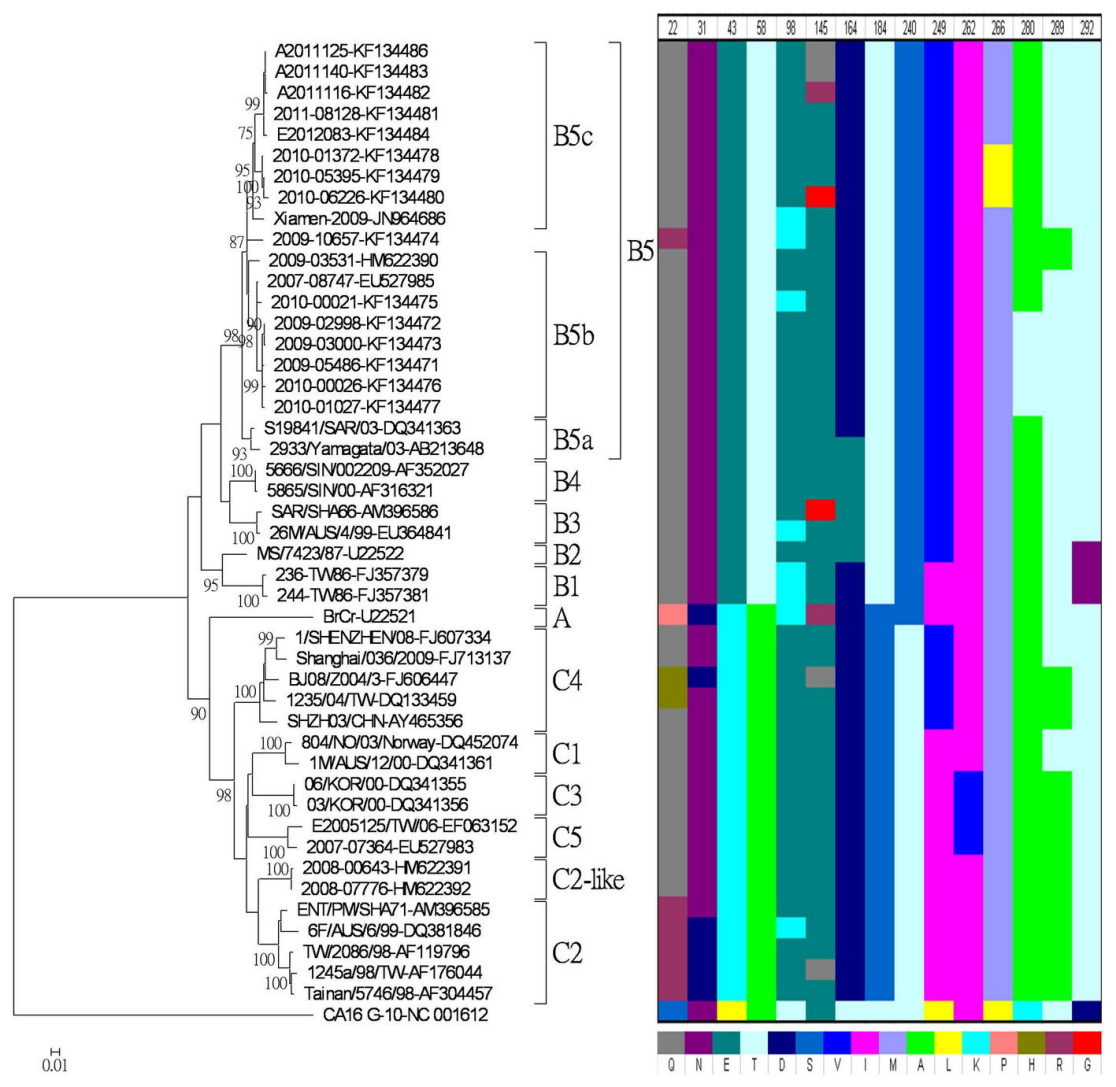
Phylogenetic analysis of enterovirus 71 (EV-71) based on complete nucleotide sequences of the VP1 gene. Phylogenetic analysis was performed based on the complete nucleotide sequences of the VP1 gene of reference strains and Taiwanese isolates. The phylogenetic tree was constructed with MEGA software by the neighbor-joining method with 1,000 bootstrap replications. Only bootstrap values of over 70% were shown. Amino acid substitutions were visualized through a proteotyping map with colored indications in single-letter abbreviations. Each column represented the amino acid position indicated with a selection criterion of 5%.

Based on the phylogenetic analysis of VP1 gene sequences (Figure 3), four different conserved amino acids were identified between the genogroups B and C of EV-71. For instance, E (glutamic acid) was at position 43, T (threonine) was at position 58, 184, and S (serine) was at position 240 for genogroup B, but these were replaced by K (lysine), A (alanine), S, and T, respectively, for genogroup C. However, no specific amino acid substitution correlated to different subgenogroups was observed among B5a, B5b and B5c isolates. 

Interestingly, there were four subgenogroup B5 isolates displaying an amino acid substitution at position 145, and three of them were isolated from the patients with acute flaccid paralysis (AFP). A2011125 and A2011140 exhibited an E145Q substitution, while A2011116 exhibited an E145R (Figure 3). 

### Neutralization antibody titers and cross-reactivity between EV-71 subgenogroups

**Figure 4:**
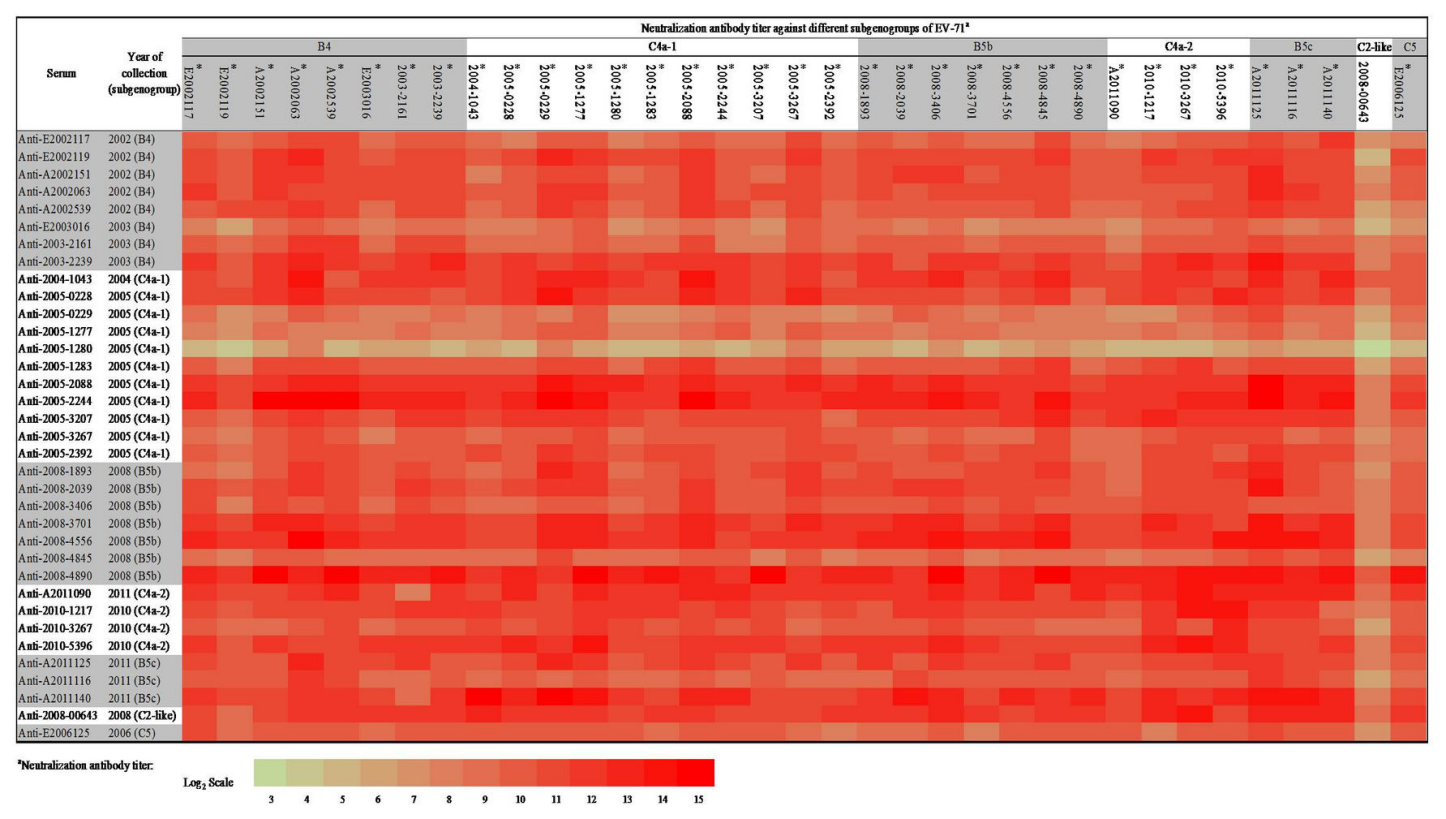
Serum neutralization antibody titers against different subgenogroups of enterovirus 71 (EV-71). The 35 serum samples were tested at two-fold serial dilutions against one hundred CCID_50_ of EV-71 viruses. The neutralization titers were defined as the highest dilution fold at which the cytopathic effects were inhibited in 50% of cells. The titer values were log_2_ transformed and were represented by different colors, as indicated by the log_2_ scale. The star symbol indicates a significant difference when compared to subgenogroup C2-like group (p<0.05).

Serum samples were collected from the patients who were infected with EV-71 subgenogroups B4, C4 (C4a-1 and C4a-2), B5 (B5b and B5c), C2-like and C5 in 2002-2003, 2004-2005, 2010-2011, 2008, 2011, 2008, and 2006, respectively (Table S1). The serum sample against C2-like virus was obtained from the C2-like-infected patient’s brother. Both patients showed enterovirus infection-related symptoms and sought medical advice. Most of the serum samples were collected during the recovery-phase (12-34 days post infection), except for two samples collected in the acute-phase (3-7 days post infection). Neutralization antibody titers against their homo-subgenogroup viruses were 1:32 to 1:8,192 (Table 1). Cross-reactive neutralizing antibody titers against EV-71 strains with different subgenogroups are shown in Figure 4. A diversity of neutralizing antibody titers was observed, with a range of 32,768 to <8. No clear pattern of antigenic variations was observed among these strains with different subgenogroups, except C2-like. Over all, the patients infected with subgenogroup B4, C4 (C4a-1 and C4a-2), B5 (B5b and B5c), and C5 had lower neutralizing antibody titers against the subgenogroup C2-like strain than against other strains (p<0.05). In addition, the C2-like infected patient had broad cross-reactivity against all strains with different subgenogroups.

**Table 1:** Neutralization antibody titers of 35 serum samples against homo-subgenogroup of enterovirus 71 (EV-71) strains.

Subgenogroup of EV-71 infection	Number of serum sample with indicated neutralization antibody titer							Total
	1:32	1:256	1:512	1:1,024	1:2,048	1:4,096	1:8,192	
B4	0	1^a^	0	2	3	1	1	8
C4a-2	0	0	0	1	0	2	1	4
C4a-1	1	1	0	1	5	1	2	11
B5b	0	0	1	0	3	1	2	7
B5c	0	0	0	0	1	1	1	3
C5	0	0	1	0	0	0	0	1
C2-like	0	0	1^b^	0	0	0	0	1

a Serum sample was collected in acute-phase (3 days post infection).

b Serum sample was collected in acute-phase (7 days post infection).

## Discussion

EV-71 infection, one of the common enterovirus infections in the Western Pacific region, was associated with HFMD, herpangina, and neurological diseases. Based on the surveillance system in Taiwan, there were several EV-71 outbreaks caused by different subgenogroups in recent years, for instance, subgenogroup B4 from 1999 to 2003, subgenogroup C4 from 2004 to 2005, and subgenogroup B5 in 2008 [14,24]. In addition, there were minor outbreaks of subgenogroup C4 from 2010 to 2011 and subgenogroup B5 from 2011 to 2012. The rapid changes of dominant subgenogroups and potential for outbreaks made it important to monitor the genetic and antigenic variations. 

In this research, we reported the genetic and antigenic diversity of EV-71 subgenogroups in Taiwan from 2009 to 2012, including the disappearance of subgenogroup C5, an epidemic of subgenogroup C4, sporadic cases of subgenogroup C2, and genetic changes of subgenogroup B5. This type of circulation was not similar to that in other countries; for example, genogroup C was the main genogroup in the United Kingdom and mainland China for years [23,33].

Subgenogroup C5 was first described in southern Vietnam in 2005, and resulted in an outbreak with neurological manifestations [11]. In 2006, subgenogroup C5 was first isolated in Taiwan [19], and it was found in a small number of cases until 2010, based on our surveillance results. Although subgenogroup C5 was not observed in Taiwan in 2011 and 2012, the possibility of C5 re-emerging could not be ignored because B5 caused outbreaks in 2008 and re-emerged in 2011-2012. 

Subgenogroup C2, the main genogroup of most EV-71 isolates from the 1998 epidemic in Taiwan, was detected in a small number of cases after 10 years. Interestingly, the C2 isolates in 2009 were clustered with those in 1998, but the isolates in 2012 were clustered with recent isolates, for example, isolates from Japan in 2010 and those from the United Kingdom in 2010. This clustering suggests that these C2 isolates evolved and spread worldwide. Additionally, the C2 isolates from Taiwan in 2012 were collected from different patients at different locations in different months, demonstrating that this subgenogroup was not a cluster event but had circulated for a period of time in a small number of cases. Moreover, a closely related phylogeny between these recent C2 isolates was suspected because of the high similarity (>98%) of the partial VP1 region.

C4, the main subgenogroup in China, has circulated and spread in recent years. There were two clusters of C4 (C4a and C4b) from 1998 to 2010, with a shift from cluster C4b to C4a in 2003–2004 [21,33]. The C4b cluster was first isolated in Taiwan in 1998, and the C4a cluster caused outbreaks in 2004-2005 [24]. In 2008, several C4 isolates (C4a) identified in Taiwan were thought to have been transmitted from China because these isolates correlated genetically with C4 strains from China but not with those isolated in Taiwan in 2004-2005 [14]. Furthermore, this C4 subgenogroup caused small outbreaks in 2010-2011 based on the present surveillance results. Additional studies are needed to clarify why subgenogroup C4 caused different epidemic patterns. For example, several large outbreaks occurred in China in specific years, but only small ones occurred in Taiwan. However, we could not exclude the effects due to the diversity of EV-71 circulation and herd immunity in Taiwan.

The subgenogroup B5 has circulated in Taiwan since 2007 and caused outbreaks in 2008, although there were two isolates sporadically detected in 2003. Towards the end of 2009, a genogroup shift (B5 to C4) and drift (B5b to B5c) of circulating EV-71 was observed. Isolate 2009-10657 was thought to be the intermediate between B5b and B5c. Although the epidemics in 2011-2012 may be partially attributed to the emerging subgenogroup B5c of EV-71, no evident divergence of antigenic relationship between subgenogroup B5b and B5c was displayed in this study. In addition to the viral factor, herd immunity and the susceptible population (infants, children, and adolescents) may be important factors in EV-71 epidemics because these groups may not have experienced the previous outbreak in 2008 [14] or produced the necessary antibodies yet. However, the genogrouping of EV-71 does not absolutely reflect the antigenicity of the strains examined [34].

An amino acid mutation at position 145 of VP1 was associated with increased virulence in mice [8]. In addition, the E145Q substitution was observed in more virulent strains [35], and this residue of genogroup B was under positive selection based on the results of evolutionary analysis with single-likelihood ancestor counting and fixed-effects likelihood methods [36]. In this study, we described three subgenogroup B5 isolates with substitutions at position 145 that were isolated from patients with AFP. These results emphasize the importance of substitution at position 145, and additional studies of receptor binding and virulence correlation with this substitution are needed. However, another genetic determinant (K244E) of mouse adaptation and virulence was not observed in this study [7].

Few studies of cross-protection among different genogroups of EV-71 were available [14,37,38]. Furthermore, the antigenicities of EV-71 strains varied based on different reports. Different genogroups displayed less than 4-fold differences in neutralizing antibody titers based on a serological survey in Japan [38]. However, antigenic differences were detected between genogroup B and C, and also between B5 and B4 viruses [36]. By using rabbit antiserum, anti-B2 could cross-neutralize B0, B1 and B2 viruses but not C1 or C2 viruses. On the other hand, anti-C1 rabbit serum could neutralize both genogroup B and C viruses [39]. Mao et al. detected different cross-neutralization capacities against different strains with the same subgenogroup (C4a) in animal sera [40]. The variations observed between different reports might be due to the different sample sizes, virus strains, sera, experimental methods, and cell lines used in the neutralization tests. However, standard protocols, including standard sera and viruses, may be used to minimize variations.

In this study, there were 35 strains and corresponding serum samples used to analyze the cross-protection and antigenic diversity among different subgenogroups (C4a, C5, B4, B5b, B5c, and C2-like) of EV-71. The subgenogroup C2-like strain exhibited evident antigenic diversity. All sera were positive against the 35 EV-71 strains, but the titers of neutralization antibody produced varied. Most of the patients infected with subgenogroup B4, C4, B5, and C5 had lower neutralizing antibody titers against the subgenogroup C2-like strain than against other strains, whereas the C2-like infected patient had broad cross-reactivity against all strains with different subgenogroups. These results were not unexpected because C2-like was a recombinant strain originating from subgenogroup C2 and B3 [14]. Since the C2-like serum was collected in the acute-phase while most other serum samples were collected in the recovery-phase, we cannot rule out the possibility that the serum collected at different stages may influence the neutralization antibody titers. However, the rabbit antisera against C2-like strain, prepared by our laboratory, were used for conducting neutralization tests for EV-71 viruses in the study, and showed similar results as using patients’ sera (Figure S1). Although C2-like viruses did not spread widely, the antigenic diversity of these viruses may lead to concern for the development of EV-71 vaccines. The key factors for selecting candidate vaccine strains include high virus titers, the ability to induce strong neutralization antibody titers, and broad cross-neutralization effects. These serum samples and the serological data obtained from the present study may be helpful to select a suitable vaccine strain and to recognize antigenic variants of EV-71. 

In conclusion, first, the genetic changes of circulating EV-71 in Taiwan in recent years were described, including the re-emergence of subgenogroup B5, the short circulation of C4, the disappearance of C5, and the sporadic occurrence of subgenogroup C2. Second, the antigenicities of different subgenogroups of EV-71 were evaluated by using serum samples obtained from patients with EV-71 infections. These results may be useful for the surveillance and prevention of EV-71, and the development of vaccines.

## Supporting Information

Table S1 **Serum samples collected from patients with enterovirus 71 (EV-71) infections.**
(DOC)

Figure S1 **Neutralization antibody titers of rabbit antisera against different subgenogroups of enterovirus 71 (EV-71).**
(TIF)
