# Supplementary material for: Antigenic and Genetic Diversity of Human Enterovirus 71 from 2009 to 2012, Taiwan
Source: PLoS One. 2013 Nov 15;8(11):e80942. doi: 10.1371/journal.pone.0080942 (PMC3858369; doi:10.1371/journal.pone.0080942)
Supplement: Table S1 — Serum samples collected from patients with enterovirus 71 (EV-71) infections. (DOC) [file pone.0080942.s001.doc]

Table S1. Serum samples collected from patients with EV-71 infections.

| Virus | Subgenogroup | Age (months) | Onset date (year/month/day) | Region | Place |
| --- | --- | --- | --- | --- | --- |
| E2002117 | B4 | 10 | 2002/3/21 | Central | Yunlin County |
| E2002119 | B4 | 30 | 2002/3/27 | Central | Taichung County |
| A2002151 | B4 | 6 | 2002/5/13 | Northern | Taipei County |
| A2002063 | B4 | 13 | 2002/4/13 | Central | Changhua County |
| A2002539 | B4 | 15 | 2002/11/6 | Central | Miaoli County |
| E2003016 | B4 | 28 | 2003/1/17 | Northern | Taipei County |
| 2003-2161 | B4 | 0 | 2001/6/5 | Central | Changhua County |
| 2003-2239 | B4 | 27 | 2003/4/2 | Central | Changhua County |
| 2004-1043 | C4a-1 | 41 | 2004/4/15 | Central | Taichung County |
| 2005-0228 | C4a-1 | 27 | 2005/4/8 | Central | Yunlin County |
| 2005-0229 | C4a-1 | 94 | 2005/4/19 | Central | Yunlin County |
| 2005-1277 | C4a-1 | 13 | 2005/5/22 | Central | Taichung County |
| 2005-1280 | C4a-1 | 23 | 2005/5/26 | Central | Taichung County |
| 2005-1283 | C4a-1 | 28 | 2005/5/27 | Central | Nantou County |
| 2005-2088 | C4a-1 | 18 | 2005/3/3 | Central | Changhua County |
| 2005-2244 | C4a-1 | 7 | 2005/4/26 | Central | Yunlin County |
| 2005-3207 | C4a-1 | 11 | 2005/5/14 | Southern | Kaohsiung City |
| 2005-3267 | C4a-1 | 56 | 2005/5/10 | Southern | Kaohsiung County |
| 2005-2392 | C4a-1 | 48 | 2005/5/20 | Central | Yunlin County |
| 2008-1893 | B5b | 21 | 2008/4/7 | Northern | Hsinchu County |
| 2008-2039 | B5b | 77 | 2008/3/17 | Central | Nantou County |
| 2008-3406 | B5b | 47 | 2008/5/31 | Central | Changhua County |
| 2008-3701 | B5b | 46 | 2008/5/3 | Southern | Tainan County |
| 2008-4556 | B5b | 26 | 2008/1/17 | Southern | Pingtung County |
| 2008-4845 | B5b | 80 | 2008/5/2 | Southern | Kaohsiung County |
| 2008-4890 | B5b | 474 | 2008/4/14 | Southern | Kaohsiung County |
| A2011090 | C4a-2 | 81 | 2011/8/18 | Northern | Taoyuan County |
| 2010-1217 | C4a-2 | 60 | 2010/5/29 | Northern | Taoyuan County |
| 2010-3267 | C4a-2 | 71 | 2010/6/14 | Southern | Tainan City |
| 2010-5396 | C4a-2 | 57 | 2010/7/30 | Northern | Hsinchu County |
| A2011125 | B5c | 41 | 2011/11/12 | Southern | Tainan City |
| A2011116 | B5c | 24 | 2011/10/4 | Central | Taichung City |
| A2011140 | B5c | 30 | 2011/11/18 | Central | Yunlin County |
| 2008-00643 | C2-like | 8 | 2008/5/1 | Northern | Taipei County |
| E2006125 | C5 | 8 | 2006/5/9 | Central | Taichung City |
