# Supplementary figures and images for: Antigenic and Genetic Diversity of Human Enterovirus 71 from 2009 to 2012, Taiwan
Source: PLoS One. 2013 Nov 15;8(11):e80942. doi: 10.1371/journal.pone.0080942 (PMC3858369; doi:10.1371/journal.pone.0080942)

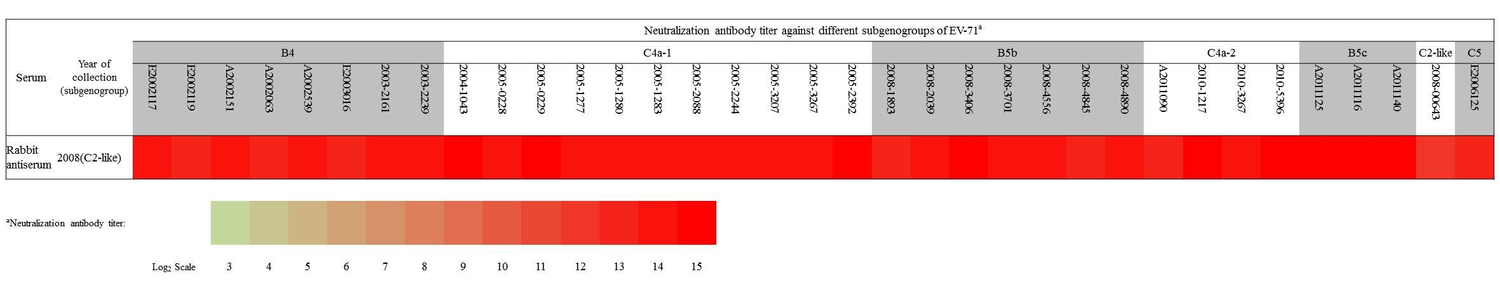

Supplement: Figure S1 — Neutralization antibody titers of rabbit antisera against different subgenogroups of enterovirus 71 (EV-71). (TIF) [file pone.0080942.s002.tif]
